# Clinical Spectrum of Near-miss Cases in Obstetrics

**DOI:** 10.7759/cureus.4641

**Published:** 2019-05-10

**Authors:** Shazia Sultana, Shahina Ishtiaque, Sundus Fareed, Samina Kamal, Zarnigah Aslam, Rubina Hussain, Sanam Lashari

**Affiliations:** 1 Obstetrics and Gynecology, Ziauddin University Hospital, Karachi, PAK; 2 Internal Medicine, Civil Hospital, Karachi, PAK

**Keywords:** maternal near-miss, obstetrics, maternal mortality, severe acute maternal morbidity, postpartum hemorrhage

## Abstract

Introduction

Near-miss obstetric cases are the ones which have survived childbirth after a life-threatening and complicated pregnancy. The aim of this study is to evaluate important characteristics and causes of near-miss cases, compare them with maternal deaths, and assess urgent interventions managing these patients.

Materials and methods

This prospective, cross-sectional study was conducted in the intensive care unit of the Obstetrics and Gynecology department. Clinical spectrum of near-miss patients was compared with that of maternal deaths. Data was entered and analyzed using Statistical Package for the Social Sciences (SPSS) Statistics for Windows, version 21.0 (IBM Corp., Armonk, NY).

Results

The incidence of near-miss events was 31.4/1,000 live births. The incidence of maternal mortality was 8.2/1,000 live births. The ratio of near-miss to maternal mortality was 3.8:1. Hemorrhage, hypertensive disorders, and puerperal sepsis were the leading causes of near-miss cases.

Conclusion

Evaluation of characteristics of near-miss cases helps in establishing severe maternal morbidity. These high-risk patients must be provided urgent interventions to prevent maternal mortality.

## Introduction

Severe acute maternal morbidity (SAMM) or near-miss obstetric cases involve women who survived a life-threatening medical condition, organ failure, or complication with respect to their pregnancy or childbirth. These cases share some of the pathophysiological and clinical characteristics with the women who succumbed to maternal mortality. Near-miss/SAMM cases are of considerable interest to obstetricians and healthcare managers. These cases help evaluate the short-comings in women healthcare facilities and identify the potential areas that need to be addressed [1].

The near-miss concept is efficient in exploring the differences, similarities, and relationships between characteristics of women who survived life-threatening pregnancy-related complications and women who actually died of them, to enable us to thoroughly gauge the quality of obstetric healthcare. This concept is equally applicable in both developed as well as developing countries [2]. Near-miss helps in connecting the dots of the cascade of events which eventually culminated in maternal death. As compared to maternal mortality, near-miss patients are more in number and provide firsthand knowledge of remote and immediate factors that may be linked to morbidity and mortality during pregnancy or within 42 days of its termination. The near-miss concept also allows initiation of awareness-based preventive programs to enhance the quality of maternal healthcare. Comparison of maternal mortality with near-miss cases helps in examining personal, social, financial, and structural predictors of maternal mortality [3].

Effective implementation of the near-miss concept will help analyze the high-risk group, plan relevant interventions for dealing with obstetric emergencies, and reinforce the entire healthcare setup for enabling favorable outcome. The aim of this study is to evaluate the characteristics of near-miss obstetric cases in a tertiary care hospital.

## Materials and methods

It was a prospective, cross-sectional study conducted in the department of Obstetrics and Gynecology at the Ziauddin University Hospital, Karachi during a one-year study period (January 1, 2018 - December 31, 2018). The study was approved by the Institutional Review Board which waived off patient consent as all information was to be collected from patient files.

Near-miss was defined, in this study, as per the World Health Organization (WHO) criteria as mentioned by Say et al. [1]. During the study period, there were 1,893 obstetric admissions out of which 163 (10.3%) were postpartum. Correspondingly, there were 1,811 live births during the study period. There were 72 (4.5%) women with SAMM; 15 (0.95%) of these died and the remaining 57 (3.6%) which survived were characterized as near-miss. All near-miss patients were admitted to the intensive care unit (ICU). Patient characteristics including mode of admission, mode of delivery, gravida, and medical co-morbidity status including miscarriages, hypertension, gestational diabetes, uterine fibroids, and fetal anomaly were recorded. Medical diagnoses were divided into four categories - intractable postpartum hemorrhage (PPH) due to retained placenta or morbidly adherent placenta, eclampsia, and puerperal sepsis. Relevant urgent interventions included hemodialysis, magnesium sulphate infusion, uterine artery re-embolization, emergency hysterectomy, and non-invasive/invasive ventilation. All parameters were compared for near-miss cases and mortal cases. Data was entered and analyzed using Statistical Package for the Social Sciences (SPSS) Statistics for Windows, version 21.0 (IBM Corp., Armonk, NY). Frequencies and percentages were calculated for categorical variables and mean and standard deviation (SD) was calculated for continuous variables.

## Results

The mean age of all women in the SAMM group was 29.67 ± 7.88 years. Among the 15 (0.95%) patients who died, five (33.3%) deaths took place either during delivery or in recovery; and the remaining 10 (66.7%) deaths were postpartum in the ICU. Of these 10 women, two (20%) were admitted after a complicated delivery from another hospital and the remaining eight (80%) had delivered in this hospital. All 57 (3.6%) near-miss cases were delivered in this hospital. The incidence of near-miss was 30.1/1,000 obstetric admissions and 31.4/1,000 live births. The incidence of maternal mortality was 7.9/1,000 obstetric admissions and 8.2/1,000 live births. The ratio of near-miss to maternal mortality was 3.8:1. The patient characteristics of both groups are compared in Table 1.

**Table 1:** Comparison of patient characteristics in the near-miss (n=57) and maternal mortality groups (n=15) ICU: intensive care unit; PPH: postpartum hemorrhage.

Characteristics	Near-miss n (%)	Mortality n (%)
Gravida		
Primagravida	14 (24.5%)	3 (20%)
Multigravida	26 (45.6%)	6 (40%)
Grand multigravida	17 (29.8%)	6 (40%)
Miscarriage		
0	35 (61.4%)	6 (40%)
1-2	20 (35.1%)	8 (53.3%)
3 and more	2 (3.5%)	1 (6.7%)
Mode of admission		
Elective	15 (26.3%)	2 (13.3%)
Emergency	42 (73.6%)	13 (86.7%)
Mode of delivery		
Assisted / Spontaneous Vaginal Delivery	23 (40.3%)	6 (40%)
Elective Caesarean	3 (5.3%)	0 (%)
Emergency Caesarean	31 (54.4%)	9 (60%)
Medical Co-morbidity		
Hypertension	11 (19.3%)	1 (6.7%)
Gestational Diabetes	9 (15.8%)	1 (6.7%)
Uterine Fibroids	5 (8.7%)	5 (33.3%)
Fetal anomaly	6 (10.5%)	2 (13.3%)
Diagnosis in the ICU		
Retained palcenta followed by PPH	18 (31.5%)	4 (26.6%)
Morbid placenta followed by PPH	8 (14.1%)	4 (26.6%)
Puerperal Sepsis	10 (17.5%)	5 (33.3%)
Eclampsia	21 (36.8%)	2 (13.3%)

In both groups, multi and grand multigravida women were more common and most were admitted through the emergency room. Among medical co-morbidities, hypertension and gestational diabetes were more common in the near-miss group. Uterine fibroids were very common in the mortality group. Overall, PPH was the most common diagnosis as seen in Table 1.

As shown in Table 1, there were 34/72 (47.2%) cases of PPH; 22 (64.7%) due to retained placenta and 12 (35.3%) due to morbid placenta. Retained placenta was more common in the near-miss group (31.5% vs. 26.6) and morbid placenta was more common in the mortality group (26.6% vs. 14.1%). There were 15/72 (20.8%) cases of puerperal sepsis. Puerperal sepsis was more common in mortality group (33.3% vs. 17.5%). There were 23/72 (32%) cases of eclampsia. Eclampsia was more common in the near-miss group than mortality group (36.8% vs. 13.3%).

As far as management was concerned, magnesium sulphate (MgSO4) infusion was given to all eclamptic patients (n=23; 32%). Hemodialysis was indicated in two (2.8%) patients, three (4.2%) patients of PPH required uterine artery re-embolization; 12 (16.7%) patients required non-invasive ventilation, and 32 (44.4%) patients required invasive ventilation. In the hemodialysis group, one patient survived and one did not (50% vs. 50%). In the MgSO4 infusion group, 21 patients survived (91.3% vs. 8.6%). In the uterine artery re-embolization group, one patient survived and two did not (33.3% vs. 66.7%). In the non-invasive ventilation group, nine patients survived and three did not (75% vs. 25%). In the invasive ventilation group, 25 patients survived and seven did not (78.2% vs. 21.8%). Emergency hysterectomy was attempted in two patients, both of whom survived (Table 2).

**Table 2:** Comparison of medical diagnosis and urgent intervention in the near-miss (n=57) and maternal mortality groups (n=15) MgSO4: magnesium sulphate; PPH: postpartum hemorrhage.

Diagnosis (n=72)	Near-miss n (%)	Mortality n (%)
Hemodialysis (n=2; 2.8%)		
Retained placenta followed by PPH	0	0
Morbid placenta followed by PPH	0	0
Puerperal Sepsis	1 (50%)	1 (50%)
Eclampsia	0	0
MgSO_4_ Infusion (n=23; 32%)		
Retained placenta followed by PPH	0	0
Morbid placenta followed by PPH	0	0
Puerperal Sepsis	0	0
Eclampsia	21 (91.3%)	2 (8.6%)
Uterine artery Re-embolization (n=3; 4.2%)		
Retained placenta followed by PPH	0	1 (33.3%)
Morbid placenta followed by PPH	1 (33.3%)	1 (33.3%)
Puerperal Sepsis	0	0
Eclampsia	0	0
Emergency obstetric hysterectomy (n=2; 2.8%)		
Retained placenta followed by PPH	0	0
Morbid placenta followed by PPH	2 (100%)	0
Puerperal Sepsis	0	0
Eclampsia	0	0
Non-Invasive Ventilation (n=12; 16.7%)		
Retained placenta followed by PPH	6 (50%)	1 (8.3%)
Morbid placenta followed by PPH	3 (25%)	1 (8.3%)
Puerperal Sepsis	0	1 (8.3%)
Eclampsia	0	0
Invasive Ventilation (n=32; 44.4%)		
Retained placenta followed by PPH	12 (37.5%)	2 (6.3%)
Morbid placenta followed by PPH	4 (12.5%)	2 (6.3%)
Puerperal Sepsis	9 (28.1%)	3 (9.3%)
Eclampsia	0	0

## Discussion

The incidence of maternal mortality is relatively lower as compared to near-miss cases (1:3.8). Previous history of miscarriage, uterine fibroids, and puerperal sepsis were more commonly associated with maternal mortality. MgSO4 infusion for eclamptic patients and assisted ventilation were crucial in saving maternal life.

This study highlights crucial similarities and differences in the near-miss and mortality group. It has assessed the clinical spectrum of these patients in much depth. However, the study has its short-comings too. Incidence of antepartum hemorrhage was not taken into account. The study findings cannot be inferred for all obstetric patients, but is only specific for high-risk patients which already fall into the WHO near-miss criteria.

This study revealed the incidence of near-miss to be 30.1/1,000 obstetric admissions and 31.4/1,000 live births which is comparably higher than the regional literature. In recent Indian studies, near-miss rate was 20.6/1,000 obstetric admissions [4] and 17.8/1,000 live births [2]. The ratio of maternal near-miss to mortality was reported to be 5.6:1 and 2.6:1 [2,4], as compared to 3.8:1 in this study. In an Ethiopian study, it has been reported to be 8.01/1,000 live births [5]. In a Pakistani study, near-miss rate was 52.2/1,000 live births and mortality ratio was 2.9/1,000 live births. The ratio of maternal near-miss to mortality was reported to be 17.7:1 [6].

The core obstetric complications predisposing pregnant women to near-miss events are almost always similar. The major chunk is formed by hemorrhagic disorders which may be antepartum, peripartum, or postpartum. Pregnancies complicated with hypertension-related disorders (eclampsia) and disorders of morbid placenta become more prone to obstetric hemorrhage. In this study, 45.6% of near-miss cases were caused by PPH and 37% by hypertensive disorders. In comparison, the literature also reports hemorrhage and hypertensive disorders to be the major predictors of near-miss cases as well as maternal mortality [5-7].

Puerperal sepsis has been an unresolved problem in the obstetric healthcare system of developing countries for a long time now. Sepsis alone has been responsible for as many as 30% of maternal deaths in low-to-middle income countries [8-9]. It is the third leading cause of maternal mortality and near-miss events after hemorrhage and hypertensive disorders [2,7].

Some pregnancy-related complications leading to high-risk childbirth are almost unavoidable. The benefit of evaluating near-miss events in depth is that the records of these patients and the hindrances they had to witness can help in creating safer and more approachable obstetric healthcare for future patients. Some of these factors may be associated with things lacking at the patient’s end such as desire for home delivery to maintain tradition, inadequate antenatal care, non-compliance with healthcare practitioner’s advice, disbelief in modern medicine, and others. Some factors are associated with delay in reaching a tertiary care institution due to longer distances, lack of transport or funds. Factors related to health system include delay in providing immediate relief and/or referral, lack of adequate intensive care facility, well-trained staff, and others [7,10].

## Conclusions

Obstetric emergencies demand prompt life-saving measures. Accepting the concept of near-miss and identifying the clinical characteristics of these patients is a substantial step towards preventing maternal mortality. Combating these issues at the level of primary care facilities has become essential. Evaluating patients for risk factors and providing high-risk and SAMM patients utmost intensive care can further decrease the ratio of maternal mortality. In order to reduce the incidence of near-miss cases, it is important to address women at basic levels including awareness about antenatal compliance, hygienic deliveries in proper healthcare facilities, and birth spacing.
